# Effect of cryopreservation on A172 and U251 glioma cells infected with lentiviral vectors designed for CRISPR/Cas9-mediated aquaporin-8 knock-out

**DOI:** 10.1371/journal.pone.0263162

**Published:** 2022-03-04

**Authors:** Hao Zhang, Shujuan Zhu, Yu Xing, Qian Liu, Zhen Guo, Ziling Cai, Zihao Shen, Qingqian Xia, Huajun Sheng

**Affiliations:** 1 Department of Human Anatomy, College of Basic Medicine, Chongqing Medical University, Chongqing, China; 2 Department of Forensic Medicine, Chongqing Medical University, Chongqing, China; West China Hospital, Sichuan University, CHINA

## Abstract

Among the three existing targeted gene editing technologies, zinc-finger nucleases, transcription activator-like effector nucleases, and clustered regularly interspaced short palindromic repeats-CRISPR-associated 9 (CRISPR-Cas9), the latter is widely used owing to its simplicity, efficiency, and low cost. Here, we routinely infected A172 and U251 cells with lentiviral vectors, in which aquaporin-8 (AQP8) was knocked out using CRISPR/Cas9. Our results indicated that cryopreservation did not significantly alter the viral infection efficiency, but influenced AQP8 expression in the infected cells at both protein and mRNA levels compared with the non-cryopreserved samples. Further, AQP8 expression at protein and mRNA levels in recovered cryopreserved infected cells did not significantly differ from those in the blank and negative controls, indicating that the lentivirus was still infectious at low temperatures. However, it failed to release the *AQP8*-targeting guide RNA in the infected cells, or the guide RNA was released, but underwent changes that caused it to malfunction in the cells with CRISPR/Cas9-mediated *AQP8* knock-out. Our findings possibly provide some insights into the reliability of lentiviruses as CRISPR/Cas9 vectors.

## 1. Introduction

The clustered regularly interspaced short palindromic repeats-CRISPR-associated 9 (CRISPR/Cas9) gene editing technology, which is widely used, is a convenient and efficient strategy for precisely and site-specifically modifying specific gene sequences of interest [1–3]. Additionally, cryopreservation is a means by which cells can be temporarily stored at low temperatures to prevent sustained growth, and reportedly, various types of cells transfected with specific plasmids or infected with specific viruses can be cryopreserved for later use [4]. The initial objective of this study was to infect glioma cell lines with AQP8 knock out so as to explore the effect of AQP8 on their proliferation. Thus, we constructed an aquaporin-8 (AQP8) knock-out viral vector using CRISPR/Cas9 and thereafter, used it to infect A172 and U251 cells. However, we incidentally observed that cryopreservation was unfavorable for infected cells with inhibited AQP8 expression; thus, it might not be applicable to all cell types.

## 2. Methods

### 2.1 Cell lines and groups

A172 and U251 glioma cell lines were infected with a lentivirus-carrying CRISPR/Cas9. Thereafter, we investigated whether AQP8 knock-out had different effects on cells with and without cryopreservation. We established two control groups, i.e., a blank (cells without viral infection) and a negative control (cells infected with an empty vector). Further, we established five experimental groups. The following five experimental groups were also established for each cell line: non-cryopreserved A172 and U251 cells infected with a CRISPR/Cas9-carrying lentivirus and cultured under normal conditions and A172 and U251 cells infected with a CRISPR/Cas9-carrying lentivirus and cryopreserved for 48 h, and 1, 2, or 4 weeks.

### 2.2 Construction and identification of the dual lentiviral-vector CRISPR/Cas9 system

An *AQP8*-targeting single guide (sg)RNA sequence was designed. Based on this sequence, single-stranded DNA oligos were synthesized and purified via polyacrylamide gel electrophoresis (Shanghai Genechem, Shanghai, China), and thereafter annealed to form double-stranded DNA fragments with sticky restriction sites at the 5′ and 3′ ends that were later ligated to the Lenti-sgRNA-tag, GV371 (U6-sgRNA-SV40-EGFP). Further, TOP10 competent cells were transformed using the ligation product, and positive clones were confirmed using the colony polymerase chain reaction (PCR) and sequenced to obtain a lentiviral knock-out plasmid expressing sgRNA (5’-TGGTGATGCTCCTCCCGTAC-3’). The CRISPR/Cas9 lentiviral vector was Lenti-cas9-puro. Further, the virus was sequenced by Shanghai Genechem.

### 2.3 Cell culture and viral infection

#### 2.3.1 Cell culture

Glioma A172 and U251 cells (Shanghai Cell Bank of Chinese Academy of Sciences, Shanghai, China) were cultured in complete Dulbecco’s Modified Eagle’s Medium (Gibco Laboratories, Gaithersburg, MD, USA) containing 10% fetal bovine serum (FBS) (Cellcook Biotech, Guangzhou, China) and supplemented with 5 mg/mL of penicillin-streptomycin (NCM Biotech, Newport, RI, USA). After ten passages, cells in a good growth state were removed for lentivirus infection, and after three passages, cells revived after cryopreservation were tested using western blotting (WB) and reverse-transcriptase quantitative PCR (RT-qPCR). The optimal viral titer for infection was ≥ 1 × 10^8^ transduction units/mL, determined using a conventional plaque assay.

#### 2.3.2 Evaluation of viral infection rate

Glioma A172 and U251 cells with good growth status were selected one day before viral infection and spread evenly on six-well plates until they reached 20–30% confluence. On the first day of infection, Lenti-cas9-puro was added to the corresponding groups of cells until the desired viral titer was attained (multiplicity of infection = 20). Thereafter, the resulting mixture was cultured routinely for 3 days, followed by puromycin screening to select cells for further infection with Lenti-sgRNA-tag (multiplicity of infection = 20). Precisely 48 h later, green fluorescent protein (GFP) expression was monitored via fluorescence microscopy. Cells were counted separately in bright and fluorescent fields (both at 50× magnification). Further, the viral infection rate, calculated as the ratio of cells in the fluorescent field to those in the bright field, was determined as > 80%, which was sufficient for subsequent experimentation.

### 2.4 Cryopreservation and recovery

#### 2.4.1 Cryopreservation

Infected cells were washed twice with phosphate-buffered saline, suspended in trypsin (1 mL), and incubated in T25 cell culture flasks at 37°C for 1 min to detach them from the flasks. Thereafter, complete medium (1 mL) was added to stop digestion, and the suspended cells were pelleted in 15-mL tubes via centrifugation at 800 rpm for 5 min. The resutling cell pellets (20 × 10^4^ cells/mL) were then mixed uniformly with a mixture of dimethyl sulfoxide and FBS at a ratio of 1:9, which served as a cryopreservative, rapidly transferred into disposable cryopreservation tubes, cooled at -20°C for 2 h, and stored overnight at -80°C. Finally, they were placed in liquid nitrogen for 48 h, or 1, 2, or 4 weeks.

#### 2.4.2 Recovery

Cryopreserved cells were transferred rapidly from liquid nitrogen into a water bath until the tube temperature reached 37°C. Thereafter, cell suspensions in 15-mL centrifuge tubes were separated via centrifugation at 800 rpm for 5 min and the supernatant was discarded. The resulting cell pellets were resuspended in complete medium and cultured routinely in T25 flasks. After resuscatation, the cells grew well, showing normal cell body morphology and protuberance. No significant difference was observed between the proliferation abilities of the freeze-preserved and non-freeze-preserved cells.

### 2.5 WB analysis

Cells were harvested and lysed in a lysis buffer containing protease and phosphatase inhibitors, both from Beyotime Biotechnology (Shanghai, China), followed by centrifugation to remove cell debris. The obtained lysates were then mixed with sodium dodecyl sulfate protein loading buffer, and the proteins were separated via sodium dodecyl sulfate polyacrylamide gel electrophoresis, and thereafter, transferred to polyvinylidene difluoride membranes. Non-specific protein binding on the membranes was blocked for 20 min using a blocking solution (Beyotime Biotechnology), after which the blots were incubated with 1:1000-diluted anti-AQP8 (Abcam, Cambridge, UK) and anti-β-actin (Cell Signaling Technology, Danvers, MA, USA) antibodies at 4°C overnight, followed by incubation with a 1:5000-diluted horseradish peroxidase-conjugated secondary antibody (Earthox, San Francisco, CA, USA) for 1 h. Proteins of interest were then visualized using an ultra-sensitive enhanced chemiluminescence color developing solution (4A Biotech, Beijing, China).

### 2.6 RT-qPCR

The Total RNA extracted from each group of cells using an RNA extraction kit (Omega Bio-Tek, Norcross, GA, USA) were reverse transcribed into first-strand cDNA as described by the manufacturer (Toyobo, Osaka, Japan) and amplified via RT-PCR using a LightCycler system (Roche Diagnostics, Basel, Switzerland) with SYBR Primescript RT-qPCR kits. The sequences of the upstream and downstream primers (5′→3′) (Sangon Biotech, Shanghai, China) were as follows:

AQP8, TGCCATCAATGAGAAGACAAAG and ATCTCCAATGAAGCACCTAATG; β-actin, AGAAAATCTGGCACCACACCT and GATAGCACAGCCTGGATAGCA. Further, the PCR conditions were as follows: 95°C for 5 min; followed by 35 cycles of 95°C for 30 s, 56°C for 30 s, and 72°C for 45 s. The collected data were analyzed using the 2^-ΔΔCt^ method, and *actb* was used as the internal reference gene.

### 2.7 Statistical analyses

All data, which were analyzed using SPSS software version 19.0 (IBM, Armonk, NY, USA), were presented as means ± standard deviation. Two-group comparisons were realized by performing Student’s *t*-test, while multiple-group comparisons were realized by performing one-way analysis of variance. All the experiments were performed in triplicates.

## 3. Results

### 3.1 Virus construction and sequencing

**Fig 1:**
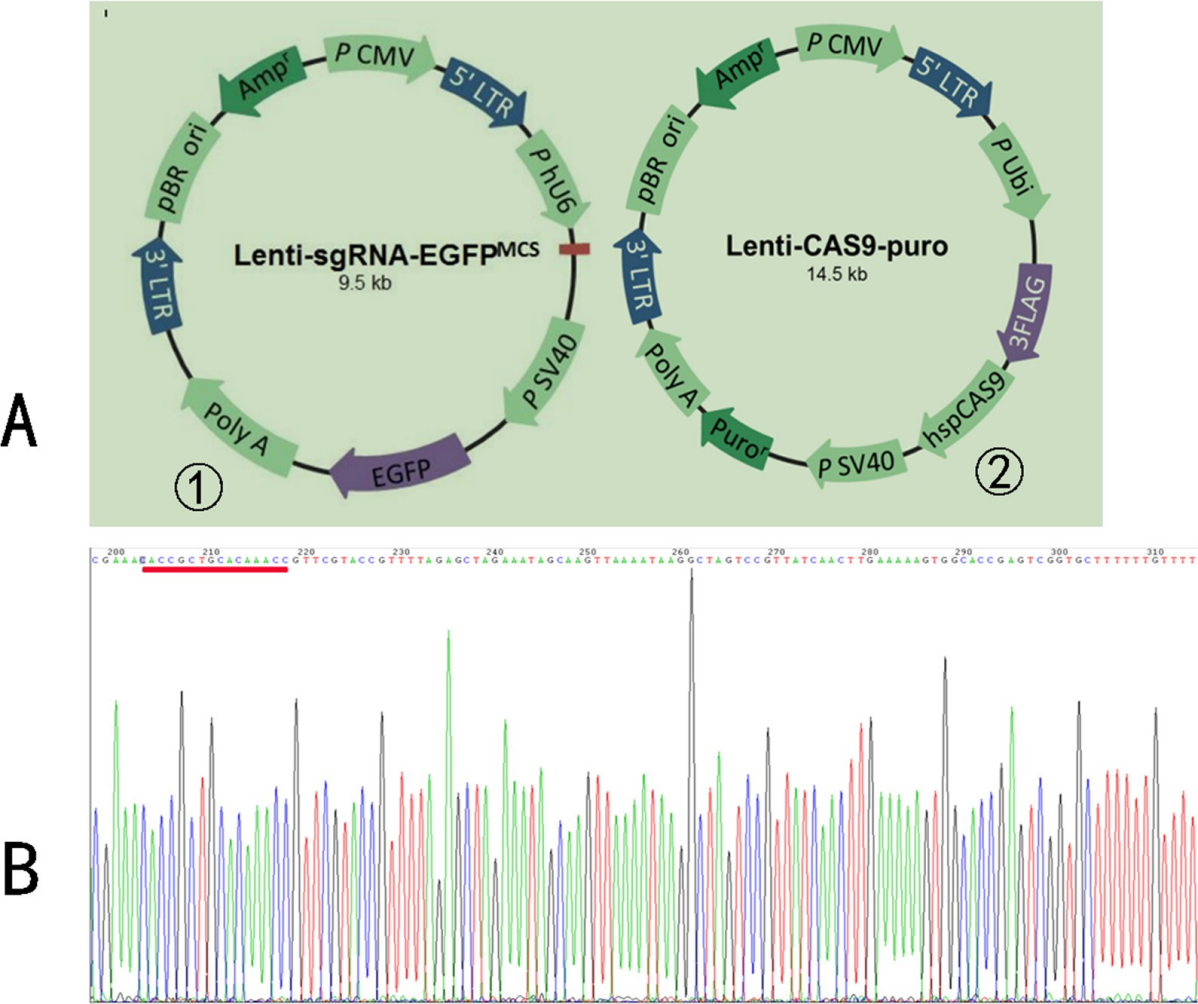
Vector GV371 and lentivirus. **A,** Vector GV371 with components linked in order of (a) U6-sgRNA-SV40-EGFP and (b) Lenti-cas9-puro. **B,** sgRNA Sequence.

Fig 1A shows the construction of the lentiviral vector Lenti-sgRNA-tag that was used to target the target gene sequence, as well as the lentiviral vector Lenti-cas9-puro that was used to knock-out AQP8. The presence of the target gene in the vectors was confirmed via sequencing (Shanghai Genechem). Fig 1B shows the sgRNA sequence, underlined in red.

### 3.2 Viral infection rate of cryopreserved versus non-cryopreserved glioma A172 and U251 cells

**Fig 2:**
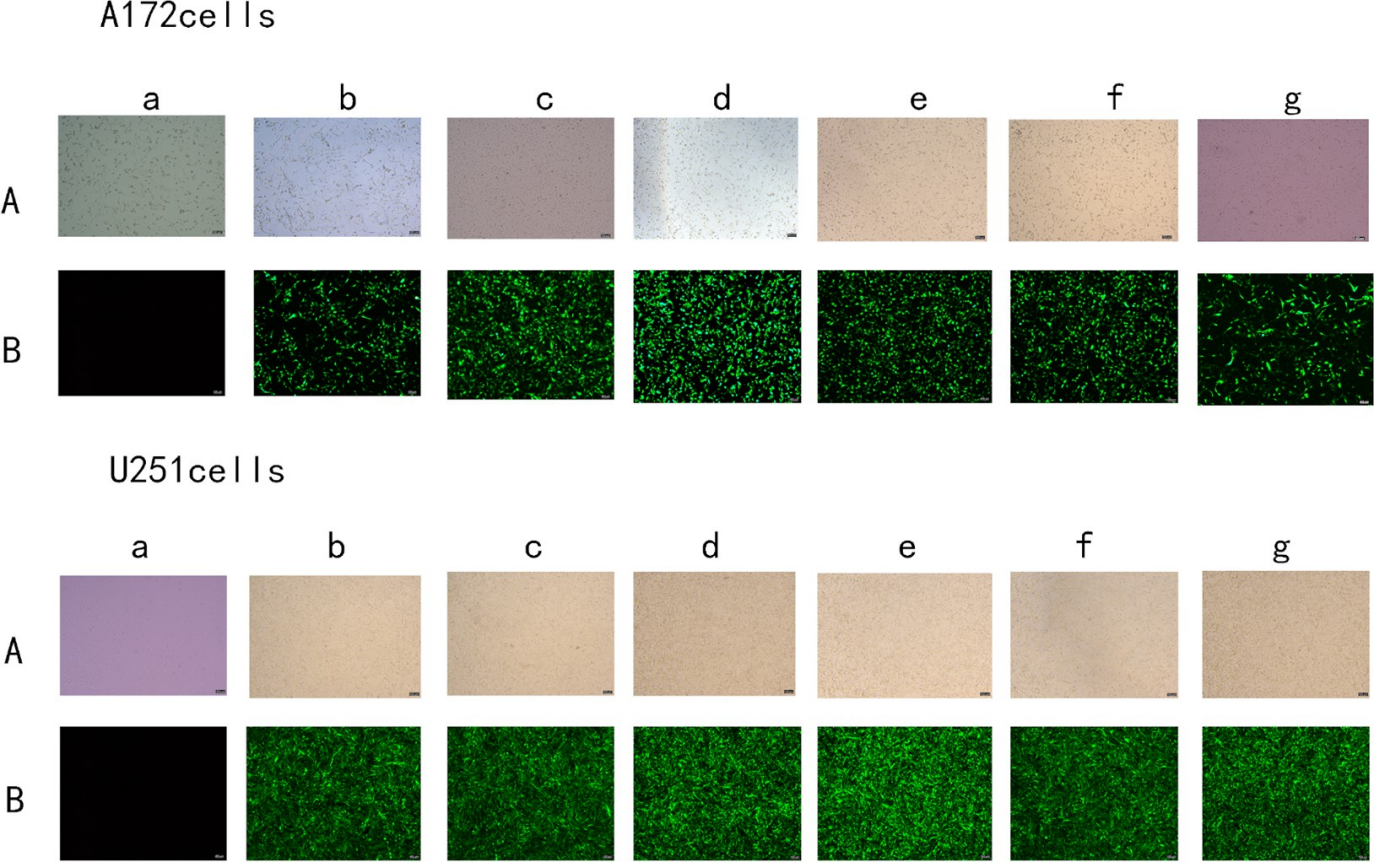
Viral infection efficiency assessed via microscopy using green fluorescent protein fluorescence. **A,** Bright field. **B,** Infected cells in the fluorescent field. Blank control (a), negative control (b), non-cryopreserved cells with *AQP8* knocked down (c), cells infected and then cryopreserved for 48 h (d) and 1 (e), 2 (f) and 4 (g) weeks.

We counted cells with and without cryopreservation in bright and fluorescent fields, and thereafter determined the degree of viral infection in the A172 and U251 cells using the fluorescent to bright field data ratio (Table 1). The GFP expression and viral infection rates corresponding to the A172 and U251 AQP8 knocked-down cells with and without cryopreservation did not differ significantly from those corresponding to the negative control cells (Fig 2).

**Table 1:** Viral infection rates. Data are shown as means ± standard deviation (SD).

Cells	Control	Negative control	Knocked down not cryopreserved	Cryopreserved 2 days	Cryopreserved 1 week	Cryopreserved (2 weeks)	Cryopreserved (4 weeks)
A172 (X ± SD)%	0.0	94.0 ± 0.1	94.8 ± 0.2	93.4 ± 0.1	91.5 ± 0.3	92.8 ± 0.4	92.4 ± 0.3
U251 (X ± SD)%	0.0	91.7 ± 0.2	94.3 ± 0.4	92.3 ± 0.3	91.1 ± 0.3	90.9 ± 0.2	93.5 ± 0.1

The different groups showed no differences in infection rates.

### 3.3 AQP8 expression in virally-infected A172 and U251 cells with and without cryopreservation

**Fig 3:**
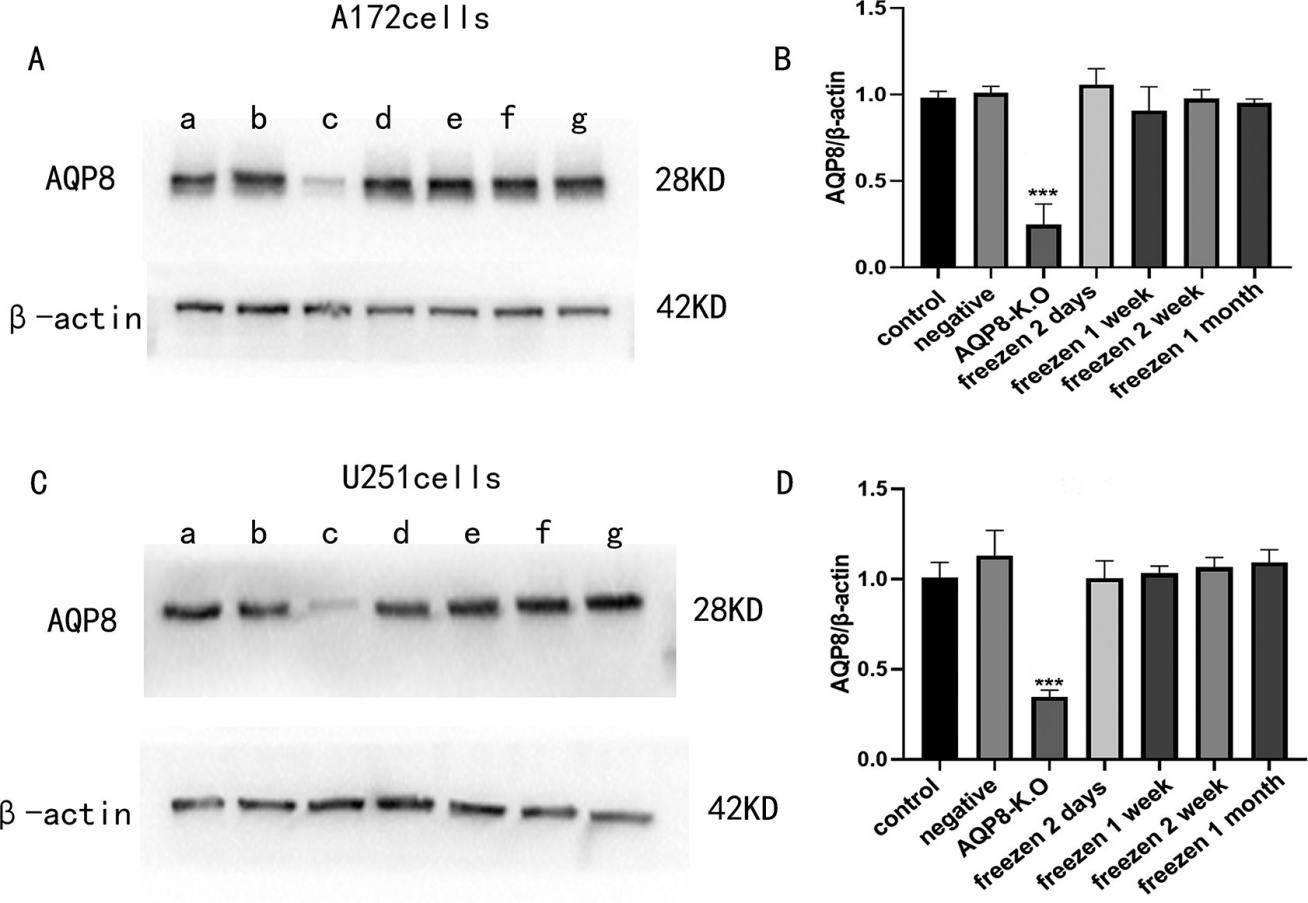
Western blots of AQP8 protein expression in lentivirus-infected A172 and U251 cells with or without cryopreservation. **A,** Changes in the AQP8 protein band in A172 cells. **B,** Comparison of the AQP8 expression levels corresponding to different groups of A172 cells. **C,** Changes in the AQP8 protein band in U251 cells. **D,** Comparison of the AQP8 expression levels corresponding to different groups of U251 cells. Blank control (a), negative control (b). Non-cryopreserved cells with AQP8 knock-out (c). Cells infected and then cryopreserved for 48 h (d) and 1 (e), 2 (f), and 4 weeks (g). *P < 0.001, non-cryopreserved cells with AQP8-knock-out vs. the other six groups.

WB (Fig 3) revealed significantly lower AQP8 expression levels (gray values) in A172 and U251 cells with AQP8 knock-out that were not cryopreserved than in the blank and negative controls (*P* < 0.001). Further, the expression level of AQP8 in infected cryopreserved and recovered cells did not differ significantly from the levels corresponding to the two controls, regardless of the duration of cryopreservation.

### 3.4 Detection of *AQP8* mRNA expression in A172 and U251 cells with and without cryopreservation using RT-qPCR

**Fig 4:**
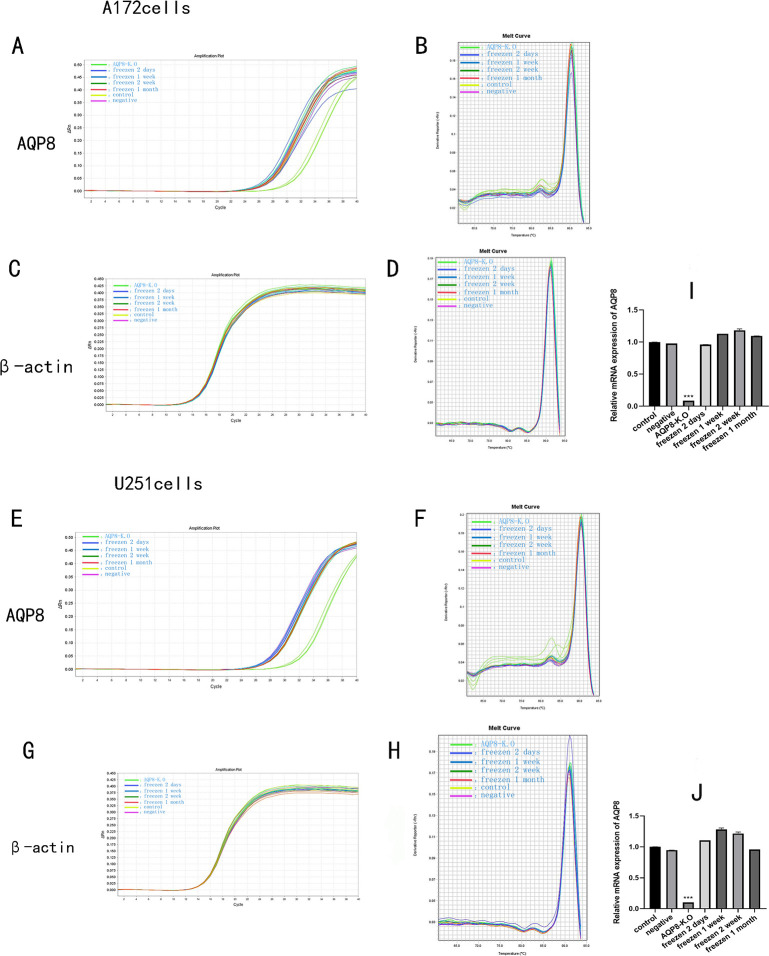
Detection of *AQP8* mRNA expression in cryopreserved vs. non-cryopreserved cells using RT-qPCR. **A,** Amplification and **B,** dissociation curves of *AQP8* mRNA in A172 cells. **C,** Amplification and **D,** dissociation curves of *actb* mRNA in A172 cells. **E,** Amplification and **F,** dissociation curves of *AQP8* mRNA in AU251 cells. **G,** Amplification and **H,** dissociation curves of *actb* mRNA in U251 cells. Relative expression level of *AQP8* mRNA in **I,** A172 and **J,** U251 cells. *P <0.001, cells with AQP8-knock-out without cryopreservation vs. the other six groups.

Fig 4 shows significantly lower *AQP8* mRNA expression in A172 and U251 cells with AQP8 knock-out without cryopreservation than in the blank and negative controls (*P* < 0.001). Further, *AQP8* mRNA expression did not differ significantly between infected, cryopreserved, and recovered cells compared with the two control groups, regardless of the duration of cryopreservation.

## 4. Discussion

In this study, the CRISPR/Cas9 gene editing technology was used to construct two AQP8 knock-out viruses, GV371 (U6-sgRNA-SV40-EGFP) and Lenti-cas9-puro (Puro represents puromycin used for screening uninfected cells). The success of the construction was confirmed via sequencing (Fig 1). Thereafter, U251 and A172 cell lines were infected with the contructed AQP8 knock-out viruses and cultured routinely for the observation of the effect of AQP8 on glioma growth and cell proliferation. However, while using cryopreserved cells, we accidentally observed certain phenomena. First, the stable cells were frozen for 48 h, 1 week, 2 weeks, and 1 month and then resuscitated for culturing. The experimental results showed that the virus infection efficiency remained above 90% compared with that corresponding to the inactive storage or negative control groups (Table 1). Further, as shown in Fig 2, no abnormal changes occurred, indicating that, with the extension of freezing time, the viruses remained in the cells and could replicate. However, WB and RT-qPCR results obtained during the assessment of viral infection (Figs 3 and 4) showed that, in stably infected cells without cryopreservation, the expression of AQP8 was inhibited at both mRNA and protein levels; this was consistent with our expectation. Second, cells stably infected with the AQP8 knock-out virus were frozen for different durations (48 h, 1 week, 2 weeks, and 1 month). After resuscitation, GFP expression in the stably infected cells did not show any significant change. Further, the AQP8 mRNA and protein expression levels in the recovered cells were not significantly different from thase corresponding to the cells in the control group, suggesting that the sgRNA targeting the *AQP8* sequence could not achieve target recognition or that the Cas9 protein could not cut the *AQP8* sequence. However, it was unclear whether the viruses lost their ability to release the specific *AQP8*-targeting sequence in cells after cryopreservation or whether the sequence was released; nonetheless, their original function was lost.

Mammalian cell lines have become important tools for a wide range of *in vitro* biomedical, biotechnological, and cancer studies [5, 6]. The sustained proliferation and growth of cultured cells are often temporarily inhibited for subsequent studies. Additionally, cell samples can be stored for a long time in a liquid nitrogen-cooled environment via cryopreservation. Dimethyl sulfoxide mixed with FBS at different ratios can also serve as a cell cryopreservation medium to decrease the freezing point of the intracellular milieu. Moreover, slow freezing allows water to slowly permeate cells; this reduces ice crystal formation and thus avoids cell damage. Further, under cryopreservation, the biological metabolism of living cells is significantly reduced, and enzymatic activity as well as chemical reactions come to a stop. This meets the experimental requirement for the temporary inhibition of cell growth. Furthermore, cell culture technology has matured and is now widely applied. Here, we confirmed the reliability of a culture technology by routinely culturing U251 and A172 cell lines to meet our experimental requirements.

Applications of lentivirus-based siRNA and CRISPR/Cas9 technologies have widened to include exploratory studies on cancer cell lines. These technologies rely on the ability of lentiviruses to deliver siRNA and CRISPR/Cas9 systems into specific cancer cells at a super-high infection rate, and are used to modify specific genes to achieve the over-expression or knock-out of a specific protein in cancer cells. This allows for the subsequent observation of the growth, proliferation, and invasiveness of the cancer cells after the changes in the expression level of a target protein. Here, the sgRNA system was delivered via lentiviral vectors into glioma A172 and U251 cells that had been previously infected with the CRISPR/Cas9 lentiviral vector and selected by puromycin, which led to the knock-out of *AQP8* in the two cell lines. Thus, the reliability of the recombinant virus was confirmed. Notably, *AQP8* knock-out disappeared in the A172 and U251 cells recovered after 48 h to 1 month of cryopreservation.

Presently, cryopreservation is an established conventional method for the preservation of lentivirus-infected target cell lines. For example, Chen et al. used a GILT knock-down lentivirus to infect U87 human glioma cells, followed by cryopreservation [7]. However, the present findings revealed, for the first time, that cryopreservation is not suitable for the intracellular survival of the virus targeting *AQP8*. Our findings also suggested that the *AQP8*-targeting guide RNA might be a key factor that affects lentiviral survival at low temperatures.

Additionally, the CRISPR/Cas9 technology is associated with off-target risk, i.e., the presence of a restriction enzyme that recognizes and cleaves a target site, while also cleaving the DNA sequence at a similar site [8], resulting in off-target events and uncontrollable mutations. Off-target risk is a main limiting factor associated with a wide range of CRISPR technology applications. An off-target detection technology known as genome-wide off-target analysis by two-cell embryo injection was established in 2019 [9]. However, owing to the limitations associated with our experimental conditions, we were unable to determine whether off-target events had occurred during lentiviral vector-mediated knock-out of *AQP8* in cryopreserved cells, and these issues need to be addressed in further investigations.

In summary, cryopreservation decreased the potency of an AQP8 knock-out virus in stably infected U251 and A172 cell lines. This finding might answer some questions regarding the reliability of the use of lentiviruses as CRISPR/Cas9 vectors.

## Supporting information

S1 Raw images (PDF)

## References

[1] Gupta D, Bhattacharjee O, Mandal D, Sen MK, Dey D, Dasgupta A, et al. CRISPR-Cas9 system: A new-fangled dawn in gene editing. Life Sci. 2019;232: 116636. doi: 10.1016/j.lfs.2019.116636 31295471

[2] Makarova KS, Haft DH, Barrangou R, Brouns SJ, Charpentier E, Horvath P, et al. Evolution and classification of the CRISPR-Cas systems. Nat Rev Microbiol. 2011;9: 467–477. doi: 10.1038/nrmicro2577 [Epub 2011 May 9]. 21552286 PMC3380444

[3] Ou X, Ma Q, Yin W, Ma X, He Z. CRISPR/Cas9 gene-editing in cancer immunotherapy: Promoting the present revolution in cancer therapy and exploring more. Front Cell Dev Biol. 2021;9: 674467. doi: 10.3389/fcell.2021.674467 34095145 PMC8172808

[4] Valyi-Nagy K, Betsou F, Susma A, Valyi-Nagy T. Optimization of viable glioblastoma cryopreservation for establishment of primary tumor cell cultures. Biopreserv Biobank. 2021;19: 60–66. doi: 10.1089/bio.2020.0050 33107762 PMC7892309

[5] Mo J, Anastasaki C, Chen Z, Shipman T, Papke J, Yin K, et al. Humanized neurofibroma model from induced pluripotent stem cells delineates tumor pathogenesis and developmental origins. J Clin Invest. 2021;131: e139807. doi: 10.1172/JCI139807 33108355 PMC7773354

[6] Olivo PD. Transgenic cell lines for detection of animal viruses. Clin Microbiol Rev. 1996;9: 321–334. doi: 10.1128/CMR.9.3.321 8809463 PMC172896

[7] Chen S, Wang Q, Shao X, Di G, Dai Y, Jiang X, et al. Lentivirus mediated γ-interferon-inducible lysosomal thiol reductase (GILT) knockdown suppresses human glioma U373MG cell proliferation. Biochem Biophys Res Commun. 2019;509: 182–187. doi: 10.1016/j.bbrc.2018.12.099 30587343

[8] Naeem M, Majeed S, Hoque MZ, Ahmad I. Latest developed strategies to minimize the off-target effects in CRISPR-cas-mediated genome editing. Cells. 2020;9: 1608. doi: 10.3390/cells9071608 32630835 PMC7407193

[9] Zuo E, Sun Y, Wei W, Yuan T, Ying W, Sun H, et al. Cytosine base editor generates substantial off-target single-nucleotide variants in mouse embryos. Science 2109. 2019;364: 289–292. doi: 10.1126/science.aav9973 30819928 PMC7301308

