# Supplementary material for: Effect of cryopreservation on A172 and U251 glioma cells infected with lentiviral vectors designed for CRISPR/Cas9-mediated aquaporin-8 knock-out
Source: PLoS One. 2022 Mar 4;17(3):e0263162. doi: 10.1371/journal.pone.0263162 (PMC8896708; doi:10.1371/journal.pone.0263162)
Supplement: S1 Raw images — (PDF) [file pone.0263162.s001.pdf]

A172

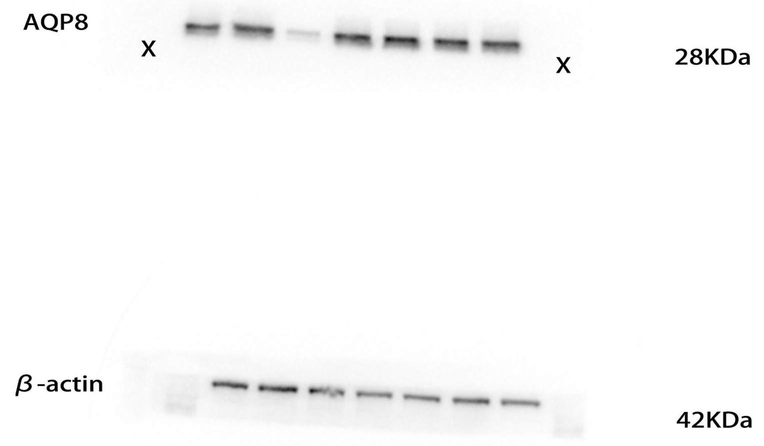

U251

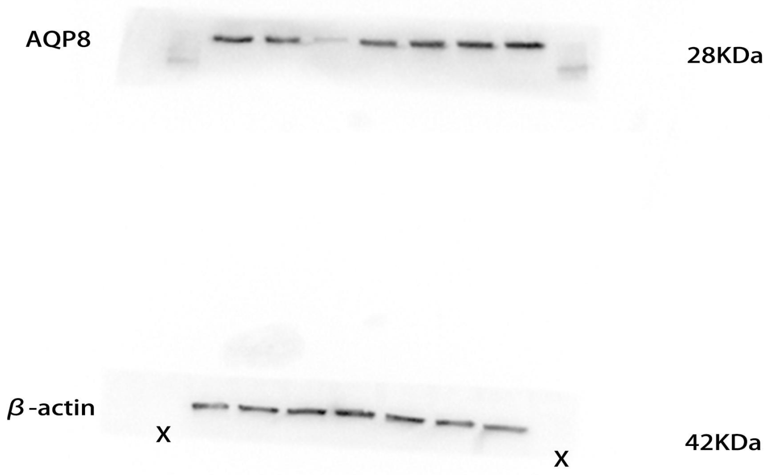

Note：The image is automatically exposed and developed by image Lab software on the wax plate，loading order as follows：control 、negative 、AQP8-K.O、 freezen 2 days、 freenzen 1 week、 freenzen 2 week、 freenzen 1 month.
